# Editorial: Mechanisms of Orofacial Pain and Sex Differences

**DOI:** 10.3389/fnint.2021.599580

**Published:** 2021-03-10

**Authors:** Sufang Liu, Phillip Kramer, Feng Tao

**Affiliations:** Department of Biomedical Sciences, Texas A&M University College of Dentistry, Dallas, TX, United States

**Keywords:** temporomandibular joint disorders, myogenic orofacial pain, oral cancer pain, postherpetic trigeminal neuropathic pain, gonadal hormones

Orofacial pain comprises multiple pain conditions that affect oral, head, face, and neck area. Such pain can be divided into different types based on its origin, including neuropathic, musculoskeletal, neurovascular, psychogenic, and idiopathic pain. Patients with orofacial pain often show sex differences with high prevalence in women. However, mechanisms underlying orofacial pain conditions and their sexual dimorphism remain elusive. With obvious differences in anatomical structures, gonadal hormones, and immune responses between males and females, it is proposed that these factors contribute to sexual dimorphism in orofacial pain. In the Special Research Topic entitled “Mechanisms of Orofacial Pain and Sex Differences,” we collected six relevant articles. These studies provide new insights into understanding orofacial pain and may aid target identification for future development of sex-specific therapies for orofacial pain.

Temporomandibular disorders (TMDs) are associated with chronic orofacial pain, which includes myofascial and/or Temporomandibular Joint (TMJ) pain. Women report TMD pain around three times more than men. Previous studies have demonstrated sex differences and sex hormonal effects in peripheral and central mechanisms of orofacial pain (Bereiter et al.) (Cairns et al., 2001, 2002; Cairns, 2007; Fischer et al., 2007; Okamoto et al., 2008; Pelegrini-da-Silva et al., 2008; Bereiter and Okamoto, 2011; Tashiro et al., 2012). Consistent with these earlier studies, a recent study (Hornung et al.) investigated the effects of ovarian hormones progesterone and allopregnanolone on estrogen-exacerbated nociception in TMJ inflammation model. Using a complete Freund's adjuvant-induced TMJ inflammation model, they observed that ovariectomized female rats show reduced nociceptive behavior in the TMJ inflammation model and daily administration with estradiol benzoate causes recurrence of the nociception in the TMJ, which is rapidly attenuated by the treatment with progesterone or allopregnanolone. This study suggests that supplying progesterone or its metabolite allopregnanolone when gonadal hormone levels get lower may be an effective approach to treating estrogen-evoked inflammatory TMJ pain. It has been demonstrated that estrogen plays a critical role in the pathogenesis of TMD pain. Besides ovarian sources, estrogen can also be synthesized in brain neurons. Interestingly, acute stimulation of nociceptors in the TMJ enhances estradiol secretion in the trigeminal subnucleus caudalis in a sex-dependent manner (Bereiter et al.). In a varicella zoster virus infection-induced postherpetic trigeminal neuropathic pain model, aromatase-derived estradiol attenuates such orofacial nociception by interacting with estrogen receptors to increase γ-aminobutyric acid-mediated neuronal inhibition in the thalamus (Kramer et al.). In addition, pituitary hormones can regulate nociceptive transmission in different orofacial pain conditions and may contribute to sex-dependent mechanisms of orofacial pain by intermodulation with gonadal hormones (Dussor et al.).

In a masseter muscle tendon ligation-induced myogenic orofacial pain model, it has been reported (Guo et al., 2011) that bone marrow stromal cells (BMSCs) produce long-lasting antihyperalgesia effect. The underlying mechanisms include their interaction with immune system and activation of endogenous opioid receptors in pain modulatory circuitry. Nuclear factor kappa B (NF-kB) is an important transcription factor for the regulation of gene expression in the immune system. The same group further investigated the role of NF-kB signaling in BMSCs-produced pain relief. They found that NF-kB signaling actually has dual roles in pain regulation: The activation of NF-kB pathway not only participates in the BMSCs-produced antihyperalgesia effect, but also contributes to the development of such orofacial pain (Guo et al.).

Additionally, in an oral cancer patient cohort, sex differences in prevalence and severity of oral cancer pain have been identified (Scheff et al.). In an immune-competent oral cancer mouse model, neutrophil-mediated endogenous analgesia occurs only in male mice (Scheff et al.), suggesting that this immune cell-mediated endogenous analgesia for treating oral cancer pain is sex-specific.

In conclusion, giving that there are sex differences in the underlying mechanisms of orofacial pain, we need to consider outcome measurements for each sex in future animal studies and clinical investigations of such pain conditions. Further understanding of sexual dimorphism in orofacial pain will help us develop sex-selective pain therapies and improve pain management for patients with chronic orofacial pain.

## Author Contributions

All authors listed have made a substantial, direct and intellectual contribution to the work, and approved it for publication.

## Conflict of Interest

The authors declare that the research was conducted in the absence of any commercial or financial relationships that could be construed as a potential conflict of interest.
